# The in vitro assessment of resin coating materials containing calcium phosphate, bioactive glass, and polylysine for glass ionomer cement restorations

**DOI:** 10.2340/biid.v12.42783

**Published:** 2025-01-14

**Authors:** Jiraporn Jiramongkhonsuk, Suyada Runglikhitcharoen, Parichart Naruphontjirakul, Piyaphong Panpisut

**Affiliations:** a Faculty of Dentistry, Thammasat University, Pathum Thani, Thailand; b Biological Engineering Program, Faculty of Engineering, King Mongkut’s University of Technology Thonburi, Bangkok, Thailand; c Thammasat University Research Unit in Dental and Bone Substitute Biomaterials, Thammasat University, Pathum Thani, Thailand

**Keywords:** Glass ionomer cements, degree of monomer conversion, resin coating, fluoride release, biaxial flexural strength

## Abstract

**Objective:**

Glass ionomer cements (GICs) require protective surface coatings to enhance their clinical performance. This study developed novel protective resin coatings for GICs containing monocalcium phosphate monohydrate (MCPM), bioactive glass nanoparticles (BAGs), and poly-L-lysine (PLS) and evaluated their physical, mechanical, and biological properties when applied to GICs.

**Materials and methods:**

Experimental resin coating materials were formulated with 5–10 wt% of MCPM, BAGs, and PLS. The degree of monomer conversion was measured usingAttenuated total reflectance-Fourier transform infrared spectroscopy (ATR-FTIR) (*n* = 6). GICs coated with the experimental materials were evaluated for biaxial flexural strength and modulus after 24 h water immersion using a universal testing machine (*n* = 8). Vickers surface microhardness up to 4 weeks of water immersion was also determined (*n* = 5). Fluoride and elemental release in water were analyzed using a fluoride-specific electrode and inductively coupled plasma optical emission spectrometry (*n* = 3). Cell viability was assessed using an MTT assay with mouse fibrosarcoma (*n* = 3). A commercial resin coating (EQUIA Forte Coat, EQ) served as control. Data were analyzed using one-way ANOVA and Tukey HSD test.

**Results:**

While EQ showed higher monomer conversion (87%) compared to experimental materials (72–74%) (*p* < 0.05), GICs coated with experimental materials demonstrated comparable strength to EQ-coated GICs. The experimental coatings exhibited similar F, Al, Na, and Si releases to EQ-coated GICs, with enhanced P release. All experimental coatings exhibited comparable cell viability (>70%) to the commercial material.

**Conclusion:**

The novel GIC protective coatings containing MCPM, BAGs, and PLS demonstrated acceptable in vitro performance comparable to commercial materials while potentially offering enhanced remineralization through increased elemental release.

## Introduction

High-viscosity glass ionomer cement (HV-GIC) is an alternative adhesive restoration for conservative load-bearing cavities [1]. Glass ionomer cements (GICs) demonstrated effectiveness in arresting caries and preventing secondary caries development [2]. The failure rate in GIC restoration was still reported to be high, especially in extensive restorations [3]. The survival rate of GICs was 64.9% at 5 years, with an annual failure rate of 10.02% [3]. However, a study demonstrated that GICs used in posterior cavity restorations exhibit a high survival rate of over 90% after 10 years [4]. The primary concern with GICs is their low mechanical properties compared with resin composites [5]. This limitation makes them susceptible to fracture, chipping, and wear under masticatory forces [6, 7].

To address these mechanical limitations, protective resin coatings have been developed. These low-viscosity materials can enhance strength [8] and reduce microleakage and wear [7, 9, 10]. The previous studies indicated that the application of coating resin increased *in vitro* flexural strength, likely due to the coating material helping seal cracks in the specimens [11, 12]. However, the application of coating resin also inhibited the release of elements such as fluoride, possibly because the coating layer acts as a physical barrier [11, 13]. A study showed that resin coatings lowered fluoride release of GICs by approximately 32–39% [11, 14], while dental adhesive coatings decreased ion release by up to 307-fold [15].

Recent developments for ion-releasing resin-based materials have focused on incorporating ion-releasing substances into resin coatings to maintain their protective benefits while enhancing remineralization potential. Surface pre-reacted glass (S-PRG) has shown promise in releasing multiple beneficial ions, including strontium, borate, fluoride, sodium, silicate, and aluminum [16]. Monocalcium phosphate monohydrate (MCPM), known for its high solubility, can enhance water absorption [17] and promote calcium and phosphate diffusion for remineralization [18]. Additionally, bioactive glass (BAG) particles offer additional remineralizing benefits by releasing calcium, fluoride, and strontium [19]. The BAG also modulated biofilm formation through environmental pH changes [20, 21].

The potential concern with resin-based materials is monomer release and biocompatibility. Inadequate polymerization can lead to monomer release, potentially shifting bacterial biofilm toward greater cariogenicity [22]. Traditional coatings containing high levels of methyl methacrylate (MMA) monomers present concerns regarding odor and potential skin irritation [23]. Poly-L-lysine (PLS) has emerged as a promising additive, demonstrating significant antibacterial action against *S. mutans* at 2 wt% concentration [24]. PLS exhibited minimal cytotoxicity and was utilized as a preservative in canned foods [25]. While PLS may increase water sorption [17] and potentially enhance ion release, higher concentrations may also affect mechanical properties [18].

This study aimed to develop MMA-free resin-coating materials to reduce skin irritation risks and unpleasant smells for patients and dental professionals during treatment. The experimental coatings incorporated MCPM, BAG, and PLS. They were evaluated for their degree of monomer conversion, biaxial flexural strength (BFS) and biaxial flexural modulus (BFM), Vickers surface microhardness, elemental release, and *in vitro* cytotoxicity. The null hypotheses proposed were as follows: (1) The experimental coatings would exhibit monomer conversion and *in vitro* cytotoxicity comparable to those of commercial materials, and (2) GIC coated with the experimental materials would not demonstrate significant differences in BFS and modulus, Vickers surface microhardness, and elemental release when compared to those coated with a commercial resin coating material.

## Materials and methods

### Materials preparation

The experimental resin coating materials were prepared by mixing the powder phase with the liquid phase. The liquid formulations contain 60 wt% urethane dimethacrylate (UDMA, Sigma–Aldrich, St. Louis, MO, USA), 36 wt% triethylene glycol dimethacrylate (TEGDMA), 2 wt% 10-methacryloyloxydecyl dihydrogen phosphate (10-MDP, Sigma–Aldrich, St. Louis, MO, USA), 1 wt% diphenyl (2,4,6-trimethyl benzoyl) phosphine oxide (TPO, Sigma–Aldrich, St. Louis, MO, USA), and 1 wt% dimethylaminoethyl methacrylate (DMAEMA, Sigma–Aldrich, St. Louis, MO, USA). The chemicals were weighed and mixed in an amber bottle using a magnetic stirrer for 30 minutes until the mixture became clear. The powder phase contains variation of silanated baroaluminasilicate glass (0.7 μm in diameter, Esstech, Essington, PA, USA), MCPM (Himed, Old Bethpage, NY, USA), BAG (King Mongkut’s University of Technology Thonburi, Bangkok, Thailand), and PLS (Handary, Brussel, Belgium).

**Figure 1:**
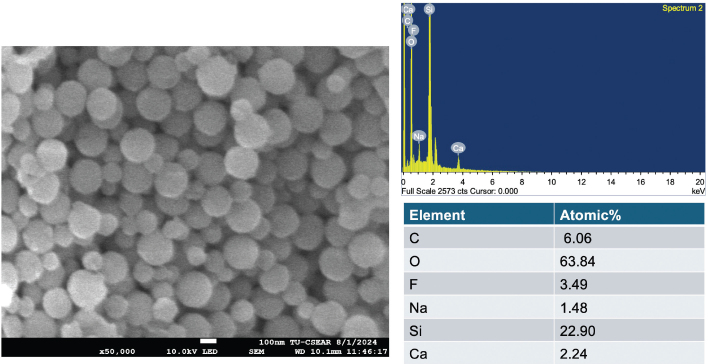
Scanning electron microscope (SEM) image of the bioactive glass particles and the elemental analysis.

The composition of the BAG used in this study was based on findings from previous research, which indicated improved elemental release and antibacterial properties when the glass was incorporated into resin-based materials [11, 26]. The BAG nanoparticles (BAG, ~200 nm diameter) were synthesized via sol-gel process and post-functionalization. For the initial synthesis, 329.2 mL of ethanol (Merck, Darmstadt, Germany), 41.1 mL of deionized water, and 4.8 mL of ammonium hydroxide (Merck, Darmstadt, Germany) were mixed in a 1 L Erlenmeyer flask and stirred at 600 rpm using a magnetic stirrer for 15 minutes. Then, 25.0 mL of tetraethyl orthosilicate (TEOS, Sigma-Aldrich, St. Louis, MO, USA) was gently added into the prepared solution and stirred for 16 to 18 h at room temperature to complete the hydrolysis and poly-condensation reactions. SiO2-NPs were collected using centrifugation at 5,000 rpm at 25°C for 30 minutes and re-suspended in deionized water. For the post-functionalization process, a total of 8.6 g of Ca(NO_3_)_2_·6H_2_O (Sigma-Aldrich, St. Louis, MO, USA), 23.1 g of Sr(NO_3_)_2_ (Merck, Darmstadt, Germany), and 0.6 g of NaF (Merck, Darmstadt, Germany) were doped into the SiO_2_-NPs using a nominal molar ratio of SiO_2_:CaO:SrO:NaF of 1.0:0.33:0.98:0.5. The particles were collected and dried at 60°C in the oven overnight to remove excess water before calcination at 680°C. The furnace temperature was increased at a rate of 3°C/minutes from room temperature to 680°C and maintained at 680°C for 3 h to remove nitrate precursors and obtain BAG nanoparticles. The final particles were washed with ethanol twice before use. The scanning electron microscope image and elemental components of the particles are provided in Figure 1.

Five different formulations of resin coating were prepared using the fixed liquid formulation but with varying concentrations in the powder phase, as shown in Table 1. These formulations were selected based on the results of similar resin-based materials tested in previous studies [11, 27]. The powder components were weighed with a 4-digit balance and hand mixed with the liquid phase at a 1:3 powder-to-liquid ratio (25% filler, 75% monomers) for 20 s in a rubber cup to produce a uniform resin paste. The paste of resin coating was then placed into a 5 mL black adhesive bottle and stored at 4°C. The commonly used commercial resin coating material (EQUIA Forte Coat, GC Corporation, Tokyo, Japan) was used as the commercial comparison (Table 2). The conventional HV-GIC used in the current study was EQUIA Forte HT Fil (GC Corporation, Tokyo, Japan).

**Table 1:** Composition of powder phase in each formulation.

Chemicals (wt%)	F1 (wt%)	F2 (wt%)	F3 (wt%)	F4 (wt%)	F5 (wt%)
Silanated baroaluminosilicate glass (diameter ~0.7 μm)	70	75	80	85	100
Monocalcium phosphate monohydrate (MCPM, diameter ~10 μm)	10	10	5	5	0
Bioactive glass (BAG, diameter ~200 nm)	10	10	5	5	0
Polylysine (PLS, diameter ~10–50 μm)	10	5	10	5	0

**Table 2:** Composition of the commercial material used in the current study.

Materials	Composition	Lot number	Supplier
EQUIA Forte HT Fil	Fluoroaluminosilicate glass, polyacrylic acid, surface-treated glass	2302091	GC Corporation, Tokyo, Japan
EQUIA Forte Coat (EC)	25–50% methyl methacrylate (MMA), camphorquinone, colloidal silica, urethane dimethacrylate, phosphoric acid ester monomer, butylated hydroxytoluene	2301231	GC Corporation, Tokyo, Japan

### Degree of monomer conversion

The degree of conversion (DC) was measured using ATR-FTIR (*n* = 6). The coating materials were placed on the ATR diamond, which was covered by a metal ring with dimensions of 0.5 mm thickness and 0.6 mm diameter, along with an acetate sheet. Light curing was performed on the top surface for 20 s using an LED curing light an LED light-curing unit (wavelength 380–515 nm, 1,500 mW/cm^2^, Eighteeth, Changzhou Sifary Medical Technology, Jiangsu, China). FTIR spectra were obtained from the bottom surface with a resolution of 4 cm^–1^ and 8 scans. The spectral range recorded was from 700 to 4,000 cm^–1^. The DC was then determined using the following equation:


$$\text{DC}=\frac{100{\text{(A}}_{\text{0}}{\text{-A}}_{\text{t}}\text{)}}{{\text{A}}_{\text{0}}}$$ Equation 1

In this equation, ΔA_0_ and ΔA_t_ represent the peak height of the C-O stretching vibration [28, 29] of the methacrylate group at 1,320 cm^–1^ above the background level at 1,335 cm^–1^, measured before curing and at the time *t* after initiating curing, respectively.

### BFS and BFM

**Figure 2:**
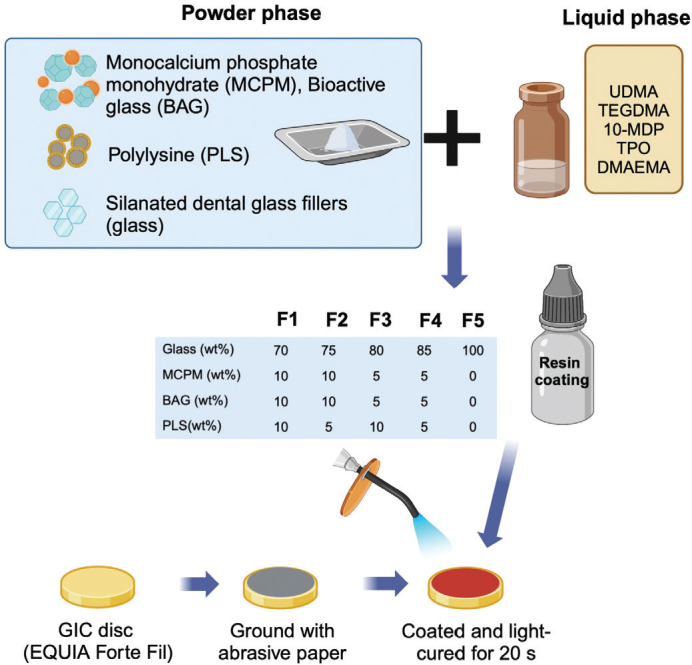
Illustration of the coating material and specimen preparation for this study. Created in BioRender. Panpisut, P. (2023) https://BioRender.com/q45p294.

GIC capsules (EQUIA Forte HT Fil, GC, Japan) were mixed in an amalgamator for 10 s (CapMix, 3M, Saint Paul, MN, USA). They were injected into a metal ring of 1 mm in thickness and 10 mm in diameter (Springmasters, Redditch, UK), covered with an acetate sheet and a glass slab, and left at room temperature for 30 minutes. Then, they were removed from the ring, and one surface was ground with no. 500 silicon-carbide paper for 10 s, followed by rinsing with running water for another 10 s (Figure 2). Subsequently, the ground surface was coated with a resin coating for 5 s, covered with a glass slide, and light-cured with an LED-curing unit for 20 s. The specimens were then immersed in 10 mL of deionized water and stored at 37 °C for 24 h before the test. The specimens were then removed, their thickness measured, and placed on a ball-on-ring testing jig.

BFS and BFM of the materials were measured using a mechanical testing frame (AGSX, Shimadzu, Tokyo, Japan). The test was conducted by applying a 500 N load cell on the jig with a crosshead speed of 1 mm/minutes. The maximum failure load was recorded, and the results were calculated using the following equations:


$$\text{BFS=}\frac{\text{F}}{{\text{d}}^{\text{2}}}\left(\text{1+v}\right)\;\text{0.485In}\;\frac{\text{r}}{\text{d}}\;+0.52\;+0.48$$ Equation 2

$$\text{BFM=}\frac{\text{H}}{{\text{W}}_{\text{c}}}\text{×}\frac{{\text{β}}_{\text{c}}{\text{d}}^{\text{2}}}{{\text{q}}^{\text{3}}}$$ Equation 3

Here, F is the failure load (N), d is the thickness of the disc specimens (m), r is the radius of the circular support of the ball-on-ring testing jig (m), and *v* represents Poisson’s ratio (0.3) [29]. $\frac{\text{H}}{{\text{W}}_{\text{c}}}$ is the rate of load change relative to central deflection or gradient of force versus the displacement curve (N/m). *β*_c_ and q are the center deflection function (0.5024) and the ratio of the support radius to the specimen radius, respectively.

The fracture surface of the representative specimen from each group was coated with gold using a sputter coater (Q150R, Quorum Technologies, East Sussex, UK). The analysis of the fracture site was conducted via a scanning electron microscope (SEM, JSM 7800F, JEOL, Tokyo, Japan) equipped with energy dispersive X-ray (EDX, X‐Max 20, Oxford Instruments, Abingdon, UK). The examination was performed at an accelerated voltage of 15 kV. EDX mapping was carried out at the center of the coating resin to identify the material’s elemental composition.

### Vickers surface microhardness

Specimens were prepared following the same procedure in the BFS testing (*n* = 5). These specimens were submerged in 10 mL of deionized water for periods of 24 h and 4 weeks. At each time interval, the surface microhardness was assessed from the coated surface using a Vickers surface microhardness tester (FM-800, Future-Tech Corp, Kanagawa, Japan). A load of 300 g was applied for an indentation duration of 10 seconds, and the readings were reported as Vickers hardness number (VHN) [30]. Measurements represented the average values obtained from four areas of the surface.

### Elemental release

For fluoride release, three coated specimens were placed in 3 mL of deionized water at 37ºC. Fluoride concentration in the solution was measured at 24 h and after 1, 2, 3, 4, and 5 weeks. At each interval, specimens were removed and placed in fresh deionized water. A calibration curve was determined using standard fluoride solutions of 1, 10, 100, and 1,000 ppm. The collected storage solution was mixed with TISAB II (Orion ionplus, Thermo Scientific, Waltham, MA, USA) at a 1:10 ratio. Fluoride concentration was measured using the specific fluoride electrode (Orion Versastar Pro, Thermo Scientific, Waltham, MA, USA).

To examine the release of elements such as aluminum (Al), phosphorus (P), calcium (Ca), silicon (Si), and strontium (Sr), separate disc specimens (*n* = 3) were prepared and placed in tubes containing 5 mL of deionized water. They were incubated at 37ºC for 4 weeks. Following the immersion period, the element concentrations in the storage solution were measured with inductively coupled plasma optical emission spectrometry (ICP-OES, Optima 8300, PerkinElmer, Waltham, MA, USA). Calibration for this analysis was performed using the environmental standard containing 26 components (CPA Chem, Bogomilovo, Bulgaria). The wavelengths and detection ranges employed for Al, Ca, P, Si, and Sr were 396 nm (0.1–20 ppm), 317 nm (0.1–50 ppm), 213 nm (0.5–20 ppm), 251 nm (0.1–50 ppm), and 460 nm (0.1–50 ppm), respectively.

### Cytotoxicity

The cytotoxicity analysis of extracts from disc specimens was performed according to the previous study (*n* = 3) [31]. The coating materials were placed in a metal circlip (0.5 mm thick and 6 mm in diameter), covered with acetate sheets and glass slides and then light-cured from the top surface for 20 seconds. They were sterilized by UV irradiation for 30 minutes on both the top and bottom sides. The samples were incubated in a supplemented Dulbecco’s modified Eagle medium (DMEM, Gibco, Thermo Fisher Scientific, Grand Island, NY, USA) for 5 days at 25ºC. The diluted extracts were used to culture L-929 mouse fibrosarcoma cells (8,000 cells per well) for 72 h at 37°C in an atmosphere of 5% CO_2_. This was compared to a blank control with a plain culture medium and a positive control using Doxorubicin. Cell viability was assessed using an MTT assay (Invitrogen, Thermo Fisher Scientific, Grand Island, NY, USA), incubating for 30 minutes at 37°C before the reaction was stopped with dimethyl sulfoxide (DMSO, Gibco, Thermo Fisher Scientific, Grand Island, NY, USA). Absorbance measurements were taken at 570 nm and 650 nm, and relative cell viability was calculated against the control. The entire experiment was conducted in triplicate. The results were expressed as relative cell viability (%) compared to the control, calculated using the following equation [32, 33]. This test was performed in triplicate.


$$\text{Relative cell viability=}\frac{\text{OD}\;\text{of}\;\text{the}\;\text{test}\;\text{group}}{\text{OD}\;\text{of}\;\text{the}\;\text{control}}\text{×100}$$ Equation 4

### Statistical analysis

Data were analyzed using Prism 10 (GraphPad Software, San Diego, CA, USA). Values are presented as mean and SD. Data normality was tested using a Shapiro-Wilk test. Normally distributed data for the degree of monomer conversion, BFS/BFM, Vickers surface microhardness, and fluoride release were analyzed using one-way ANOVA followed by the Tukey HSD test. For elemental release and cell viability results, the data were analyzed using the Kruskal‑Wallis followed by the Dunn test. Additionally, a factorial analysis was employed to determine the effect of additive concentration on the outcomes measured. Sample size estimation was conducted using G*power software, based on results from a previous study [11], to achieve a power greater than 0.95 and alpha = 0.05 for a one-way ANOVA.

## Results

### Degree of monomer conversion

**Figure 3:**
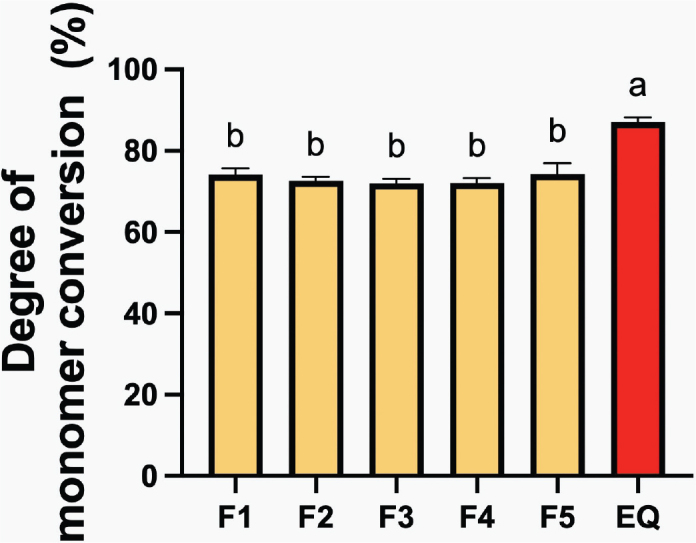
Degree of monomer conversion of resin coating materials after 40 seconds of light-curing. Error bars represent SD (*n* = 5). Identical letters represent *p* > 0.05.

The highest conversion degree of the resin coating was observed with EQUIA Forte Coat (EQ) (87.04 ± 1.11%) (Figure 3). This value was significantly greater than that of F1 (74.09 ± 1.51%, *p* < 0.01), F2 (72.51 ± 0.96%, *p* < 0.01), F3 (71.92 ± 1.18%, *p* < 0.01), F4 (72.00 ± 2.53%, *p* < 0.01), and F5 (74.31 ± 2.53%, *p* < 0.01). However, there were no significant differences among the various experimental materials (*p* > 0.05). The effect of increasing additive concentration was minimal.

### BFS and BFM

**Figure 4:**
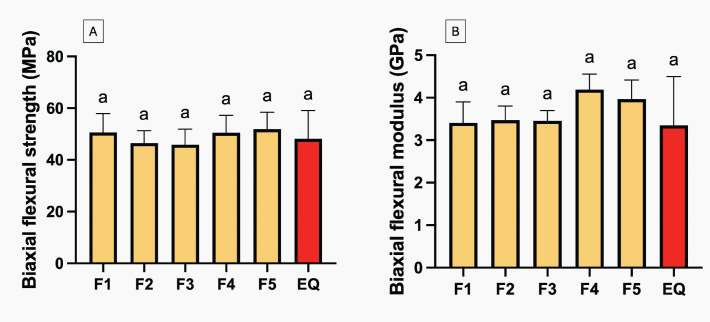
Biaxial flexural strength (A) and biaxial flexural modulus (B) of glass ionomer cements (GICs) coated with experimental and commercial resin coating materials after immersion in deionized water for 24 h. Error bars are SD (*n* = 6). Identical letters represent *p* > 0.05.

The highest BFS (Figure 4A) recorded was for GIC coated with F5 (51.9 ± 7.9 MPa), which was comparable to that of other materials (*p* > 0.05). There were no significant differences between the experimental materials. Regarding BFM (Figure 4B), F4-coated GICs exhibited the highest value (4.2 ± 0.4 GPa), while EQ had the lowest (3.4 ± 1.4 GPa); however, all results were similar (*p* > 0.05). The addition of the additives showed minimal impact on BFS, but the additives (MCPM + BAG) and PLS slightly lowered BFM by approximately 9.4 ± 9.2% and 9.6 ± 9.1%, respectively.

**Figure 5:**
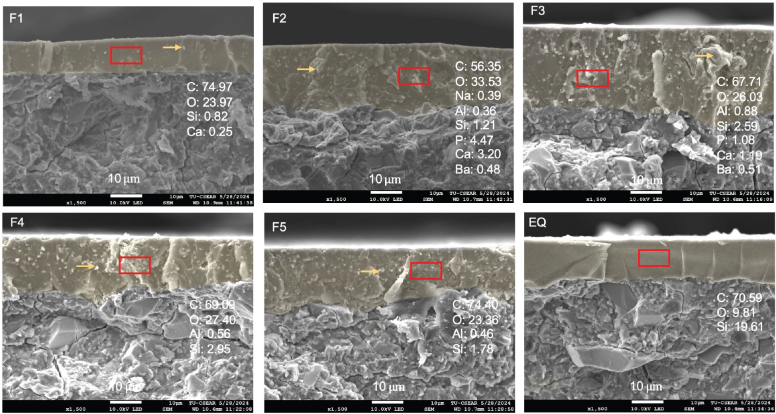
Fracture surface of tested specimens from each group. Highlighted in yellow is the resin coating. Elemental analysis using energy dispersive X-ray (EDX) mapping was done on the resin coating (rectangular area). Arrows show the filler components of the experimental materials.

The coated layer on the fracture surface of the tested specimen (Figure 5) from the experimental group was less smooth and homogeneous compared to EQ. Various elements such as Ca, Al, Si, and P were detected from F2 to F3.

### Vickers surface microhardness

**Figure 6:**
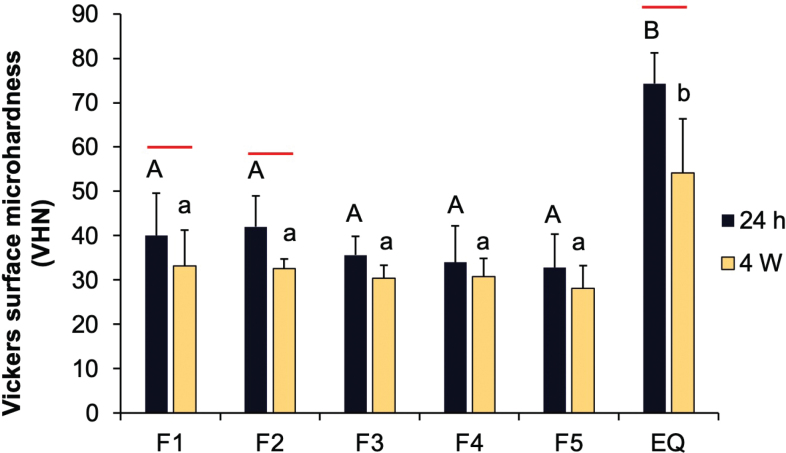
Vickers surface microhardness of glass ionomer cements (GICs) coated with experimental and commercial resin coatings after immersion in deionized water for 24 h and 4 weeks. Error bars indicate SD (*n* = 6). Identical uppercase and lowercase letters show *p* > 0.05 at 24 hours and 4 weeks, respectively. The red lines indicate *p* < 0.05 for comparisons within the same materials.

The highest Vickers surface microhardness at 4 weeks was observed in GICs coated with EQ (54.2 ± 13.9 VHN) (Figure 6). This value was significantly higher compared to F1 (33.2 ± 9.2 VHN, *p* = 0.0027), F2 (32.6 ± 2.5 VHN, *p* = 0.0020), F3 (30.4 ± 3.4 VHN, *p* = 0.0006), F4 (30.7 ± 4.7 VHN, *p* = 0.0008), and F5 (28.1 ± 5.8 VHN, *p* = 0.0002). No significant effect was found from increasing the concentration of reactive fillers.

Significant decreases in surface microhardness after 4 weeks of immersion were observed for GICs coated with F1 (*p* = 0.0425), F2 (*p* = 0.0462), and EQ (*p* = 0.0180). Adding more MCPM and BAG increased surface microhardness by approximately 18 ± 9% at 24 hours, but this effect was minimal after 4 weeks.

### Elemental release

**Figure 7:**
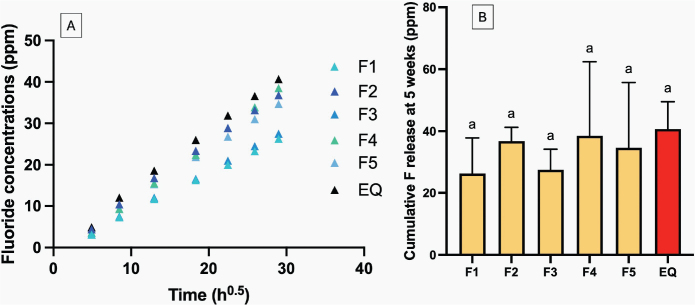
(A) The cumulative release of fluoride for up to 5 weeks. Error bars from the scatter plot were removed to aid visualization. (B) The cumulative release of fluoride at 5 weeks. Errors bars are SD (*n* = 3). Identical letters indicate *p* > 0.05.

The release of fluoride in all coated GICs demonstrated the linear release with the square root of time throughout the experimental period of up to 5 weeks (Figure 7A). The highest fluoride release at 5 weeks was observed with EQ (40.7 ± 3.5 ppm), comparable to other materials (*p* > 0.05) (Figure 7B). The increase in concentration of MCPM and BAG showed minimal effect on fluoride release. The rising concentration of PLS reduced the cumulative fluoride release by approximately 28 ± 10%.

**Figure 8:**
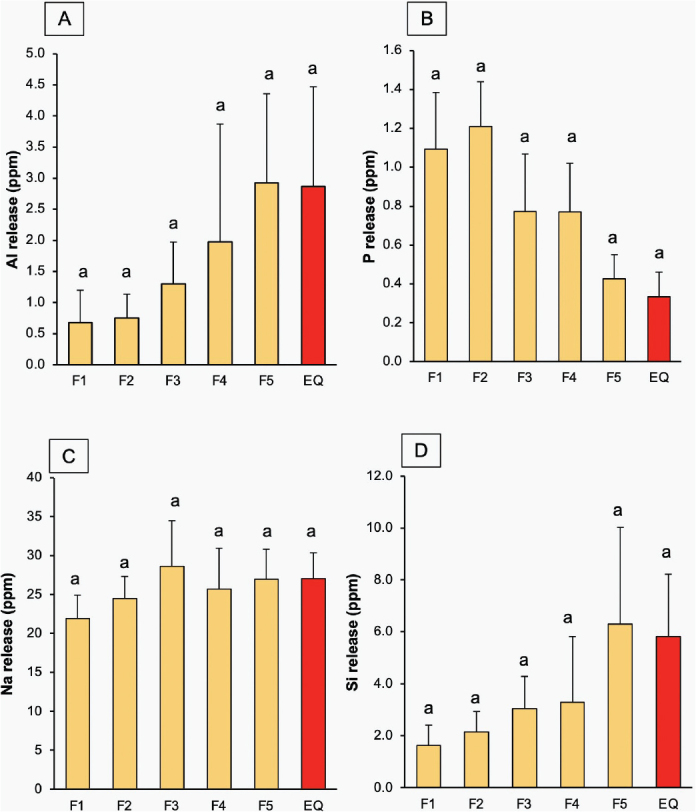
(A–D) The release of Al, P, Na, Si for 4 weeks. Errors bars are SD (*n* = 3). Identical letters indicate *p* > 0.05.

For Al release (Figure 8A), the GICs coated with EQ exhibited the highest level (2.9 ± 1.6 ppm), but this was not significantly different from that of other materials (*p* > 0.05). The increase in MCPM and BAG concentration exhibited minimal effect on the release level of Al. For P release (Figure 8B), F1 showed the highest release (1.1 ± 0.3 ppm), but this was comparable to other materials (*p* > 0.05). Factorial analysis revealed that increasing MCPM and BAG resulted in ~54 ± 33% rise in P release, while an increase in PLS showed minimal effect. The release of Na (Figure 8C) was highest in F3 (28.6 ± 5.9 ppm), while the lowest value was detected with F1 (21.9 ± 3.0 ppm). However, these values were not significantly different from other materials (*p* > 0.05). The effect of additives on the elemental release was minimal. For Si release (Figure 8D), F5 exhibited the highest value (2.9 ± 1.4 ppm), whereas F1 showed the lowest value (0.75 ± 0.4 ppm). However, the releases from all materials were statistically similar (*p* > 0.05).

### Cytotoxicity

**Figure 9:**
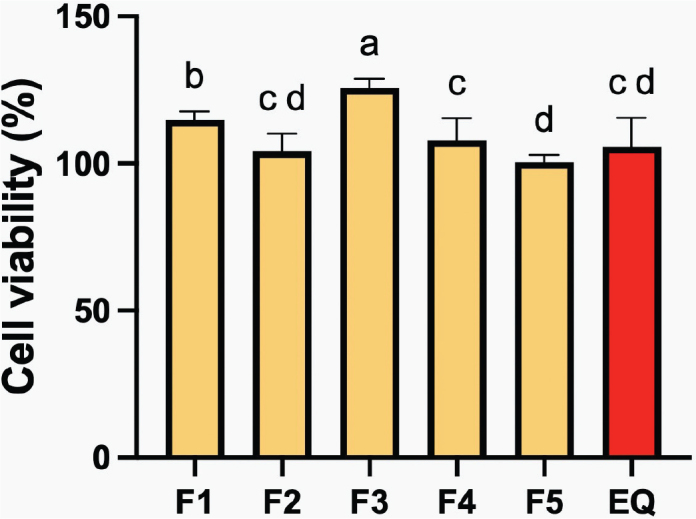
The percentage cell viability after exposure to extract of the resin coatings. Errors bars are SD (*n* = 3). Identical letters indicate *p* > 0.05.

The highest cell viability compared to the blank control was detected with F3 (108.0 ± 3.0%), while the lowest was seen with F5 (100.4 ± 1.0%) (Figure 9). The cell viability of F3 was significantly higher than that of the other groups (*p* < 0.05). The cell viability of EQ (105.7 ± 4.0%) was similar to that of F4 (108.0 ± 3.0%) and F5. Increasing levels of MCPM and BAG reduced cell viability by about 6 ± 2%, while an increase in PLS enhanced cell viability by approximately 12 ± 1%.

## Discussions

The current study prepared and investigated the *in vitro* performance of experimental resin coatings containing MCPM, BAG, and PLS. The results showed that while the commercial resin coating material exhibited higher DC than the experimental materials, both demonstrated comparable cytotoxic effects. Therefore, the first null hypothesis was partially rejected. The second null hypothesis was accepted, as GIC specimens coated with the experimental materials showed comparable performance to those coated with commercial products in BFS, BFM, surface microhardness and elemental release.

The high degree of monomer conversion in EQ could be attributed to using low molecular weight and short molecular chains such as MMA, which generally show a high conversion rate [34]. The experimental resin coating includes UDMA as the base monomer, which exhibited a high glass transition temperature (–35.3˚C). This might contribute to the lower monomer conversion observed in the experimental material compared with the commercial material [35]. Additionally, the presence of fillers could affect the DC by promoting light scattering [36], thereby hindering light penetration to the bottom side of the material and leading to a lower DC. The DC of EQ in the current study (~87%) was also higher than that reported in the previous study (~66%) [11], which could be attributed to using a thinner metal circlip in the FITR test (0.5 mm versus 1 mm), allowing light to pass through the bottom surface more easily.

The experimental material showed a conversion level between 72 and 74%, aligning with the reported range (40–75%) for resin-based materials used in restorations [37]. Achieving a high level of polymerization of resin coatings in the current study is desirable to help minimize the risk of toxic monomer release, which could potentially cause irritation or toxic effects [38, 39]. The high DC obtained from the experimental material could be attributed to using a high level of TPO, which showed higher efficacy in DC than camphorquinone (CQ) [40]. High DC and low risk of monomer leaching were also expected to ensure high strength [41], good color stability [42], and a lower risk of biofilm formation [43].

The findings of the present study suggest that incorporating hydrophilic fillers into the resin coatings had minimal impact on the strength of the coated GICs. This observation can be explained by the fact that the material’s overall strength primarily depends on the bulk structure of the GIC itself [44]. As the same GIC material was used, thus consistent strength values should be observed across all experimental groups. The coating layer may primarily function by occluding surface cracks and voids [45].

Significant reductions in surface microhardness of the coating materials were observed at 4 weeks for formulations containing high concentrations of BAG and MCPM, as well as for EQ. In the experimental coatings, the hydrophilic reactive fillers may promote water sorption or release over time [19], leading to the plasticization of the polymer matrix. For EQ, its polymer network, formed by low molecular weight MMA monomers, may be more flexible and suitable for water sorption [46] that plasticizes the polymer matrix [47]. As water absorption in resin-based materials is a continuous process [48], longer immersion periods may be necessary to assess its mechanical performance. Additionally, the water sorption of the resin coating should be tested in future work.

The release of fluoride from all GICs coated with experimental resin coating or commercial material demonstrated that the release followed the diffusion control mechanism as it is directly proportional to the square root of time [49]. This study also indicated that the level of additives added to the resin coating showed minimal effects on the elemental releasing properties of the materials, especially Ca and Sr, which could play a role in promoting mineralization [50]. The initial Sr levels might be low as indicated by the absence of detection in the EDX analysis of the glass. Therefore, further research may be required to optimize and enhance the additive concentrations, which facilitate the release of key ions for mineralization or biofilm modulation. A previous study that developed a similar formulation of resin-coating but containing PRG ionomer fillers showed comparable results, where a low level of Ca was detected, but a high level of P was released [11]. It was suggested that the released Ca might interact with fluoride, forming CaF_2_ salts, thereby leading to the failure to detect the element.

This study modified the resin coating formulation from the previous study [11] by incorporating PLS, which is known for its antibacterial properties. Higher PLS concentrations improved cell viability. PLS contains positively charged hydrophilic amino groups at pH 7 [51] The PLS may buffer the pH and reduce acidity resulting from MCPM disproportionation [52], creating a more favorable environment for cells compared to formulations with lower PLS concentrations. Increased PLS concentrations reduced fluoride release from the GICs. We hypothesize that the positively charged groups in the PLS [51] may interact with negatively charged fluoride ions, thereby reducing free fluoride availability. A limitation of the current study was the lack of investigation into the antibacterial properties of the coated GIC. Future studies should examine the effect of PLS-containing resin coatings on biofilm growth

The commercial coating material exhibited an unpleasant odor due to high concentrations of MMA monomers. However, EQ extract demonstrated no substantial cytotoxicity. This favorable outcome likely resulted from proper curing according to the manufacturer’s protocol, leading to a high degree of monomer conversion that enhanced crosslinking and reduced the monomer release [37]. Cell viability was greater than 70%, indicating low toxicity risk according to the British Standard: Biological evaluation of medical devices Part 5: Tests for in vitro cytotoxicity (ISO 10993-5:2009) [33].

This study aimed to develop MMA-free resin-coating materials to reduce the risk of skin irritation risks and unpleasant odors for both patients and dental professionals during treatment. The results indicated that the experimental materials showed comparable properties to the commercial material in terms of BFS, elemental release, and cytotoxicity. Therefore, the null hypothesis was partially accepted. Several limitations of the current study should be acknowledged. Additional tests, including biofilm modulation and remineralizing action assessments, are needed to optimize the formulation and determine appropriate reactive filler concentrations. While increasing reactive filler concentrations showed a minimal negative impact on the material’s mechanical and physical properties, higher filler levels may be required to enhance ion release for anticaries effects.

## Conclusion

The experimental resin coating, free from MMA monomers and incorporating MCPM, BAGs, and PLS, demonstrated satisfactory monomer conversion and cytotoxicity levels comparable to commercial products. GICs coated with this experimental formulation exhibited mechanical strength similar to those with commercial coatings. These findings may suggest that this material could serve as a viable alternative for coating GIC restorations.

## Data Availability

The data that support the findings of this study are available from the corresponding author upon reasonable request.

## Key messages

1. The experimental resin coating demonstrated similar physical and mechanical performance to commercial resin coating when coated on glass ionomer cements (GIC).
2. The experimental coatings maintained a similar fluoride release profile and enhanced phosphorus release for GIC through reactive fillers, potentially improving remineralization.
3. The experimental resin coatings exhibited *in vitro* biocompatibility comparable to a commercial product.
